# Smooth muscle tumors of uncertain malignant potential of the uterus: A single-center 9-year retrospective cohort study with a record-based diagnostic consistency audit

**DOI:** 10.1097/MD.0000000000051288

**Published:** 2026-10-30

**Authors:** Firat Ekmez, Numan Cim

**Affiliations:** a Department of Obstetrics and Gynecology, Haseki Training and Research Hospital, University of Health Sciences, Istanbul, Türkiye.

**Keywords:** leiomyosarcoma, mitotic count, pathology reporting, retrospective cohort, smooth muscle tumor of uncertain malignant potential, uterine neoplasms

## Abstract

Uterine smooth muscle tumors of uncertain malignant potential (STUMP) are diagnosed by integrating cytological atypia, mitotic activity, and coagulative tumor cell necrosis, yet retrospective series often rely on historical reports that document these features incompletely. This study aimed to describe an institutional STUMP cohort and to determine whether its recorded category labels were supported by the pathological variables recorded for each case. This single-center retrospective cohort study included 20 patients who underwent surgery between January 2017 and March 2026 and received an institutional diagnosis of STUMP, epithelioid STUMP, or STUMP/leiomyosarcoma (LMS) interface. In a post hoc, record-based audit, the 18 labels recorded as Criterion A, B, or C were compared with explicit operational definitions adapted from a published 3-pattern formulation and classified as discordant, indeterminate, or consistent on record. Slides were not reexamined, and original diagnoses were retained. Exact 95% confidence intervals (CIs) and 3 sensitivity analyses were calculated. The mean age was 41.6 ± 7.2 years. All patients underwent surgery for presumed leiomyoma, 11 by hysterectomy and 9 by uterus-sparing procedures. All 7 Criterion C labels were accompanied by recorded mitotic counts below 15 per 10 high-power fields (7/7; 95% CI, 59.0–100.0%). Of the 18 audited labels, 10 were discordant (55.6%; 95% CI, 30.8–78.5%), six were indeterminate, and two were consistent on record. No recorded mitotic-count range crossed an audit threshold; lower range bounds and a stricter mitotic threshold changed no audit status, whereas 14 labels were discordant when only absent atypia was accepted as nonsignificant. One STUMP/LMS interface case was subsequently managed as LMS. Sixteen patients had no recorded recurrence or progression during a median follow-up of 11 months; the outcome was unknown in 3. STUMP was not recognized preoperatively in this cohort, but without a leiomyoma comparator, no conclusion about clinical distinguishability is possible. The audit demonstrates discordance between recorded labels and recorded variables rather than diagnostic error, and the affected cases remain provisional pending slide-level gynecological pathology review. The data support no inference about recurrence risk, the safety or reproductive benefit of uterus-sparing surgery, or biomarker-based prognosis.

## 1. Introduction

Uterine leiomyomas are the most frequent neoplasms of the female reproductive tract,^[1]^ whereas uterine sarcomas are rare tumors with an aggressive clinical course.^[2]^ Between these poles lies a group of smooth muscle neoplasms that cannot be classified with confidence as benign and yet do not satisfy the criteria for leiomyosarcoma (LMS), and the fifth edition of the World Health Organization classification retains the designation smooth muscle tumor of uncertain malignant potential (STUMP) for them.^[3,4]^ The diagnostic logic of this category derives from the Stanford study of Bell, Kempson, and Hendrickson, which showed that cytological atypia, mitotic activity, and coagulative tumor cell necrosis acquire prognostic meaning only when they are assessed in combination rather than in isolation.^[5]^ In contemporary practice, these 3 variables are interpreted together with the type of necrosis, the presence of atypical mitotic figures, epithelioid or myxoid differentiation, and selected immunohistochemical findings. Because the category is defined by integration and exclusion, the diagnosis is usually established only after surgery, and the uncertainty it carries extends to surgeons, pathologists, and patients alike, most acutely when the patient is young and wishes to preserve fertility.

Published STUMP series differ considerably in their case definitions, in the duration of follow-up, and in the manner in which recurrence is ascertained, and recurrences have been documented both as STUMP and as frank sarcoma.^[6–9]^ These differences are compounded by limited diagnostic reproducibility. Centralized expert review has been advocated as a means of refining the diagnosis in retrospective cohorts,^[10]^ slide review reclassified 3 of 19 institutional STUMP diagnoses as sarcomas in 1 single-center series,^[11]^ and in another, the original diagnosis was upheld after review in 34 of 44 initially considered cases.^[12]^ It follows that a STUMP label held in an institutional archive cannot be assumed to reflect the criteria it names, and that comparisons of subtype distribution or recurrence across cohorts presuppose a correspondence between labels and underlying morphology that is seldom demonstrated. Where slides are unavailable or have not yet been reexamined, the pathological variables recorded alongside each diagnosis are the only evidence against which the label can be checked. A comparison of this kind cannot reproduce slide-level reclassification, but it can reveal where documentation fails to support the stated category and can therefore direct expert review toward the cases that require it. Ancillary markers do not resolve the problem. Individual series have proposed additional histological parameters as predictors of adverse outcome,^[13]^ whereas pooled analyses have shown that altered p53 and p16 expression, but not Ki-67, was associated with disease-free survival,^[14]^ and that atypical mitotic figures did not increase the risk of recurrence.^[15]^ Such markers supplement the reliable application of the core morphological criteria but cannot substitute for it.

The decisions that follow a postoperative STUMP diagnosis concern the extent of surgery, the method of tissue extraction, and the intensity of surveillance. Uterus-sparing management has been reported in carefully selected patients who wished to conceive,^[16–18]^ although reproductive outcomes in such series refer to pregnancies achieved after treatment, which must be distinguished from tumors removed at cesarean delivery. Occult malignancy is a further consideration: the U.S. Food and Drug Administration recommends that laparoscopic power morcellation be performed only with a tissue containment system and only in appropriately selected patients,^[19]^ and related aspects of management have been addressed in case series and in current European guidelines.^[20,21]^ Each of these decisions depends on the confidence with which the pathological category has been assigned.

The present study reports a 9-year single-center cohort from Istanbul, Türkiye, and pursues 2 objectives. The first was to describe the clinical, surgical, pathological, immunohistochemical, and follow-up characteristics of patients who received an institutional diagnosis of STUMP. The second was to determine, through a transparent record-based audit, whether the category labels recorded as Criterion A, B, or C were supported by the mitotic counts, necrosis descriptions, and atypia grades documented for the same cases, using explicitly stated operational definitions and decision rules. The audit was designed to identify documentation-level consistency, discordance, or uncertainty while preserving every original diagnosis; it involved no reexamination of slides and assigned no alternative diagnosis. By separating what the records demonstrate from what only slide review can establish, it is intended to indicate which cases can contribute to subtype comparisons and which require expert re-adjudication first. Because the cohort contains no leiomyoma comparator, the study was not designed to evaluate whether STUMP can be distinguished clinically from leiomyoma, nor to estimate the accuracy of any preoperative test.

## 2. Materials and methods

### 2.1. Study design, setting, and ethical approval

This single-center retrospective observational cohort study was conducted at the Department of Obstetrics and Gynecology, Haseki Training and Research Hospital, University of Health Sciences, Istanbul, Türkiye, and included patients treated between January 2017 and March 2026. The study protocol was approved by the Non-Interventional Clinical Research Ethics Committee of Haseki Training and Research Hospital (decision no. 140–2026, dated June 10, 2026). Because the study used only retrospective clinical and pathology records, the requirement for individual informed consent was waived in accordance with the committee’s policy, and informed consent was therefore not obtained. All data were anonymized before analysis, the study was conducted in accordance with the Declaration of Helsinki, and the report follows the Strengthening the Reporting of Observational Studies in Epidemiology recommendations.^[22,23]^

### 2.2. Case identification, eligibility, and data collection

Institutional gynecology and pathology records were searched to identify consecutive patients who underwent surgery for a presumed uterine leiomyoma or smooth muscle tumor during the study period and subsequently received an institutional pathological diagnosis of uterine STUMP, epithelioid STUMP, or STUMP/LMS interface. Patients were eligible if the smooth muscle tumor was of uterine origin, if core clinical and surgical data were available, and if the pathology record documented at least one of the following: the mitotic count, a description of necrosis, a description of atypia, or the original diagnostic category. Demographic, clinical, operative, histopathological, immunohistochemical, and follow-up data were abstracted from the institutional records. Patients with incomplete follow-up were retained in the baseline and pathology analyses but were not counted as recurrence-free when their oncological outcome was unknown. The study size was determined by the number of eligible patients treated during the study period, not by a sample-size calculation for comparative hypothesis testing. Patient names and hospital protocol numbers were removed before analysis, and the analytic case identifiers assigned in the anonymized data set were retained unchanged in all tables; these identifiers are therefore not consecutive.

### 2.3. Histopathological variables and diagnostic framework

The diagnoses and category labels analyzed in this study were retained exactly as recorded in the institutional pathology data set. For each case, the mitotic count per 10 high-power fields (HPF), the necrosis descriptor, and the atypia grade were abstracted from the historical pathology reports, and no slides were reexamined for this study. The labels Criterion A, B, and C are therefore the objects of the audit rather than diagnoses assigned by it, and they should not be read as a comprehensive contemporary classification. The conceptual basis of the framework is the Stanford approach, in which atypia, mitotic activity, and coagulative tumor cell necrosis are assessed in combination, and atypia is regarded as significant when it is moderate or severe.^[5]^ The explicit 3-pattern structure and the mitotic threshold were taken from the formulation that Ning et al reported as their application of the 2014 World Health Organization criteria, namely tumor cell necrosis without atypia and with fewer than 10 mitoses per 10 HPF; moderate-to-severe atypia without tumor cell necrosis and with fewer than 10 mitoses per 10 HPF; and at least 15 mitoses per 10 HPF without atypia or tumor cell necrosis.^[11]^ The threshold of 15 mitoses per 10 HPF is accordingly attributed to this later formulation and not to the original Stanford study.

On this basis, the operational definitions were as follows. Criterion A required diffuse moderate-to-severe atypia, fewer than 10 mitoses per 10 HPF, and no coagulative tumor cell necrosis; the requirement for diffuse atypia was retained from the investigators’ original category definition. Criterion B required coagulative tumor cell necrosis, fewer than 10 mitoses per 10 HPF, and no significant atypia. Criterion C required at least 15 mitoses per 10 HPF, no coagulative tumor cell necrosis, and no significant atypia. In the primary analysis, absent or mild atypia was treated as nonsignificant for Criteria B and C. This is an explicit adaptation of the no-atypia wording used by Ning et al consistent with the Stanford distinction between none-to-mild and moderate-to-severe atypia,^[5]^ and its influence was examined in a sensitivity analysis (Section 2.6). The 3-pattern scheme does not encompass every STUMP morphology recognized in current practice, since contemporary descriptions also include tumors with focal or multifocal atypia and borderline mitotic counts, tumors whose mitotic count cannot be established, and epithelioid and myxoid variants with their own thresholds,^[3,9]^ while uncertain types of necrosis constitute a recognized diagnostic difficulty of their own.^[12]^ Failure of a recorded label to satisfy an operational A, B, or C definition therefore does not exclude STUMP and was not interpreted as evidence of misdiagnosis. Because the microscope field area was not documented, mitotic counts were analyzed in the reported HPF units and were not converted to mitoses per mm^2^.

The audit was conceived after data abstraction and is reported as a post hoc, exploratory analysis. The 3 criterion definitions correspond to those used by the investigators when the recorded labels were first compared with the recorded variables, whereas the decision rules for ambiguous descriptors, recorded ranges, and audit status described below were formalized during the preparation and revision of this report, after the case-level variables had been examined. Neither the definitions nor the rules were therefore fixed before the case-level findings were reviewed. Once formalized, however, the rules were applied uniformly to all 18 labels recorded as Criterion A, B, or C, and the audit does not constitute a prespecified or blinded validation.

Four decision rules were applied (Fig. 1). First, mitotic counts recorded as ranges were retained in Table 3. The upper bound of each range was used in the primary analysis, and every range was examined for values lying on opposite sides of the thresholds of 10 and 15 mitoses per 10 HPF; a range spanning either threshold would have rendered the mitotic component indeterminate. Second, necrosis recorded as coagulative was provisionally accepted as satisfying the necrosis component of Criterion B. Necrosis recorded as absent or infarct-type did not satisfy it, and nonspecific, uncertain, or mixed terminology, including “present” (Turkish “var”), “necrosis,” “tumor necrosis,” “tumor necrosis?,” and mixed infarct-type and tumor necrosis, was coded as indeterminate rather than resolved by assuming the most severe interpretation. This coding does not independently verify coagulative tumor cell necrosis. Third, atypia recorded as absent or mild was classified as nonsignificant. Atypia recorded as moderate, moderate-to-severe, or severe was classified as significant, and atypia recorded as mild-to-moderate or not specified as indeterminate. Because the distribution of atypia was not recorded, the diffuse atypia required for Criterion A could not be confirmed in any case. Fourth, the case with epithelioid morphology and the case recorded as STUMP/LMS interface were described separately and excluded from the A to C audit. Each label was then classified as discordant when at least 1 recorded variable was incompatible with its operational definition, as indeterminate when no variable was incompatible but a required feature was ambiguous or missing, and as consistent on record when all required recorded features were compatible, with discordance taking precedence when both conditions applied. These statuses describe recorded labels, not final diagnoses, and no alternative diagnosis or probability of malignancy was assigned to any case.

**Table 1:** Demographic, clinical, and surgical characteristics of the cohort (N = 20).

Characteristic	Value
Age, yr; mean ± SD (range)	41.6 ± 7.2 (28–55)
Menopausal status: premenopausal/perimenopausal/postmenopausal	17 (85.0)/3 (15.0)/0
Gravidity; mean (range)	2.8 (0–5)
Parity; mean (range)	2.3 (0–5)
Nulliparous	1 (5.0); Case 25
Abnormal uterine bleeding	16 (80.0)
Pelvic pain	11 (55.0)
Sensation of a mass	2 (10.0)
Abdominal distension	1 (5.0)
Incidental diagnosis	1 (5.0)
Preoperative working diagnosis	Presumed uterine leiomyoma in all 20 cases
Imaging	Ultrasonography in all cases; MRI additionally in 1 case
Hysterectomy	11 (55.0): TAH 8; TLH 3
Uterus-sparing procedure	9 (45.0): abdominal (open) myomectomy 6; hysteroscopic myomectomy 2; myomectomy at cesarean delivery 1
Postoperative complication recorded	0
Largest tumor dimension, cm; mean ± SD (range)	6.8 ± 3.1 (1.2–12.0)

Values are n (%) unless otherwise specified; symptoms were not mutually exclusive.

MRI = magnetic resonance imaging, SD = standard deviation, TAH = total abdominal hysterectomy, TLH = total laparoscopic hysterectomy.

**Figure 1.**
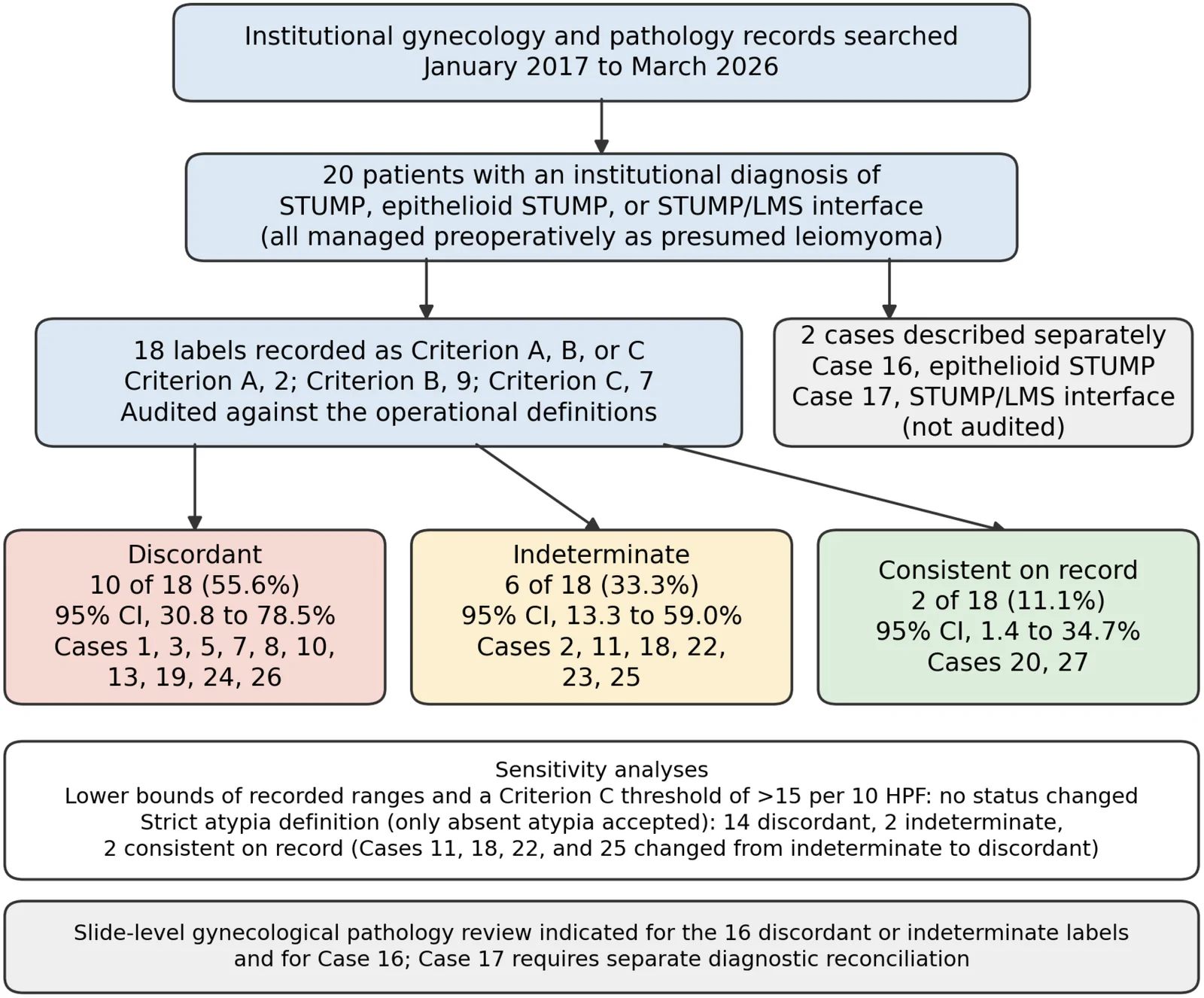
Operational definitions and decision rules of the record-based audit. (A) Operational definitions applied to the labels recorded as Criterion A, B, or C, adapted from the 3-pattern formulation reported by Ning et al ^[11]^ and grouping absent and mild atypia as nonsignificant in accordance with Bell et al ^[5]^ (B) Coding of each recorded variable as satisfying, not satisfying, or indeterminate for the relevant component of the definition. The necrosis descriptor is coded in opposite directions for Criterion B, which requires coagulative tumor cell necrosis, and for Criteria A and C, which require its absence. Terms in quotation marks are reproduced as recorded in the historical pathology reports, and no ambiguous descriptor was resolved by assuming its most severe interpretation. (C) Assignment of audit status, with discordance taking precedence over indeterminacy. The sensitivity analyses listed at the foot of the figure are described in Section 2.6. HPF = high-power field, STUMP = smooth muscle tumor of uncertain malignant potential.

### 2.4. Immunohistochemistry

Immunohistochemical results were abstracted as reported. The panel varied between cases because staining indications and institutional practice changed during the 9-year study period. The markers recorded included Ki-67, p16, p53, estrogen receptor, progesterone receptor, CD10, h-caldesmon, desmin, smooth muscle actin, fumarate hydratase, and, in individual cases, selected epithelial or melanocytic markers. Only interpretable results were summarized quantitatively, and tests documented as performed but lacking a reported result were treated as missing. Historical p53 descriptors were retained as recorded, and a result reported only as negative was not retrospectively converted into a wild-type or aberrant staining pattern. Immunohistochemical findings were summarized descriptively, and no association between any marker and any outcome was tested.

### 2.5. Outcomes and follow-up

Follow-up duration was calculated in months from the index surgery to the last documented gynecological or oncological visit. Recurrence was defined as the histologically confirmed reappearance of STUMP or LMS after the index surgery. A case that was managed as LMS shortly after the index surgery was reported as a STUMP/LMS interface event and not as a conventional recurrence of an independently verified STUMP. Patients whose recurrence or progression status was recorded as unknown were not grouped with recurrence-free patients, even when a numerical follow-up interval was available. Because the follow-up interval and the oncological status were recorded as separate variables, the availability of one was not taken to imply the other. The absence of recorded recurrence was treated as a descriptive observation. No recurrence rate, survival estimate, or other measure of recurrence risk was calculated because the cohort was small, follow-up was short and incomplete, and the diagnostic categories were heterogeneous.

### 2.6. Statistical analysis

All analyses were descriptive. Continuous variables were summarized as mean ± standard deviation or as median and range, and categorical variables as counts and percentages with explicit denominators. No comparative hypothesis tests, multivariable models, or biomarker prognostic analyses were performed, and missing or ambiguous pathological values were retained as missing or indeterminate without imputation. Exact binomial (Clopper–Pearson) 95% confidence intervals (CIs) were calculated for the principal audit proportions to convey their limited precision; these intervals do not account for selection bias or measurement error. Three sensitivity analyses were performed. In the first, the mitotic summaries and audit statuses were recalculated with the lower bound of each recorded range; in the second, the Criterion C threshold was changed from at least 15 to more than 15 mitoses per 10 HPF; and in the third, a strict atypia definition was applied, under which only atypia recorded as absent satisfied the no-atypia component of Criteria B and C. None of these analyses involved new patient observations or slide review. The audit classification, exact CIs, and sensitivity analyses were computed from the case-level values in Table 3 with a script written in Python (version 3.12.3) using SciPy (version 1.17.1).

**Table 3:** Case-level record-based audit of recorded category labels against recorded pathological variables (N = 20).

Case	Recorded category	Mitoses per 10 HPF	Necrosis recorded	Atypia recorded	Audit status and basis
1	Criterion B	6	Present	Moderate	Discordant. moderate atypia is incompatible with criterion B; the necrosis type is unspecified.
2	Criterion B	3	Tumor necrosis	Absent	Indeterminate. “Tumor necrosis” does not independently establish coagulative tumor cell necrosis.
3	Criterion C	4–5	Infarct-type	Mild	Discordant. The mitotic count is below the criterion C threshold.
5	Criterion C	1	Infarct-type	Mild-to-moderate	Discordant. The mitotic count is below the criterion C threshold; the atypia grade is also ambiguous.
7	Criterion A	15	Absent	Mild-to-moderate	Discordant. The mitotic count exceeds the Criterion A limit; the atypia grade is ambiguous. No alternative diagnosis assigned.
8	Criterion C	7	Absent	Mild	Discordant. The mitotic count is below the Criterion C threshold.
10	Criterion C	2	Absent	Absent	Discordant. The mitotic count is below the Criterion C threshold; the recorded features support none of Criteria A, B, or C.
11	Criterion B	2	Coagulative	Mild-to-moderate	Indeterminate. mild-to-moderate atypia cannot establish the no-significant-atypia component of Criterion B. Myomectomy at cesarean delivery.
13	Criterion A	12	Present	Absent	Discordant. The mitotic count and absent atypia are incompatible with Criterion A; the necrosis type is unspecified.
16	Epithelioid STUMP	NR	Absent	Not specified	Not audited against A–C. Epithelioid morphology recorded; mitotic count and atypia details incomplete.
17	STUMP/LMS interface	30–40	Focal/indeterminate	Moderate	Not audited against A–C. Interface case managed as LMS after the index surgery; p16 positive, Ki-67 60%.
18	Criterion B	3–5	Infarct-type + tumor necrosis	Mild	Indeterminate. Mixed necrosis terminology does not establish the necrosis component of Criterion B.
19	Criterion C	6–7	Infarct-type	Moderate	Discordant. The mitotic count is below the Criterion C threshold, and moderate atypia is also incompatible. Outcome unknown.
20	Criterion B	5	Coagulative	Absent	Consistent on record under the provisional necrosis coding.
22	Criterion B	2–3	Coagulative	Mild-to-moderate	Indeterminate. Mild-to-moderate atypia. Outcome unknown.
23	Criterion B	1	Tumor necrosis?/infiltrative necrosis?	Not specified	Indeterminate. Necrosis is uncertain, and atypia is unspecified.
24	Criterion C	7	Absent	Moderate-to-severe	Discordant. The mitotic count and significant atypia are incompatible with Criterion C. Outcome unknown.
25	Criterion B	3	Necrosis	Mild	Indeterminate. nonspecific necrosis terminology. Nulliparous patient.
26	Criterion C	1	Absent	Moderate-to-severe	Discordant. The mitotic count and significant atypia are incompatible with Criterion C.
27	Criterion B	1	Coagulative	Absent	Consistent on record under the provisional necrosis coding.

Audit status refers only to compatibility with the operational definitions in Section 2.3: discordant, at least 1 recorded variable is incompatible; indeterminate, a required feature is ambiguous or missing without a definite incompatibility; consistent on record, all required recorded features are compatible. Neither consistency nor discordance establishes a final diagnosis. For recorded ranges, the upper bound was used in the primary analysis. Analytic case identifiers from the anonymized data set were retained and are not consecutive.

HPF = high-power field, LMS = leiomyosarcoma, NR = not recorded, STUMP = smooth muscle tumor of uncertain malignant potential.

## 3. Results

### 3.1. Clinical characteristics and surgical management

Twenty patients met the eligibility criteria (Fig. 2), and their demographic, clinical, and surgical characteristics are summarized in Table 1. The mean age at diagnosis was 41.6 ± 7.2 years (range, 28–55); 17 patients (85.0%) were premenopausal, 3 (15.0%) were perimenopausal, and none were postmenopausal. The mean gravidity was 2.8 (range, 0–5), and the mean parity was 2.3 (range, 0–5). The only nulliparous patient, Case 25, was 38 years old, underwent myomectomy, and had no recorded recurrence at 48 months. Abnormal uterine bleeding was recorded in 16 patients (80.0%), pelvic pain in 11 (55.0%), a sensation of a mass in 2 (10.0%), abdominal distension in 1 (5.0%), and an incidental diagnosis in 1 (5.0%). All 20 tumors were approached clinically and radiologically as presumed leiomyomas before the definitive pathological diagnosis was available; ultrasonography was performed in every case, and 1 patient additionally underwent magnetic resonance imaging. Hysterectomy was performed in 11 patients (55.0%), 8 by total abdominal hysterectomy, and 3 by total laparoscopic hysterectomy. The remaining 9 patients (45.0%) underwent uterus-sparing procedures: six an abdominal (open) myomectomy, 2 a hysteroscopic myomectomy, and 1 a myomectomy at the time of cesarean delivery. No postoperative complication was recorded. The largest recorded tumor dimension had a mean of 6.8 ± 3.1 cm (median, 7.0; range, 1.2–12.0), and the tumors were predominantly intramural, subserosal, or mixed intramural-subserosal; one was submucosal, and one presented as a prolapsing vaginal myoma.

**Figure 2.**
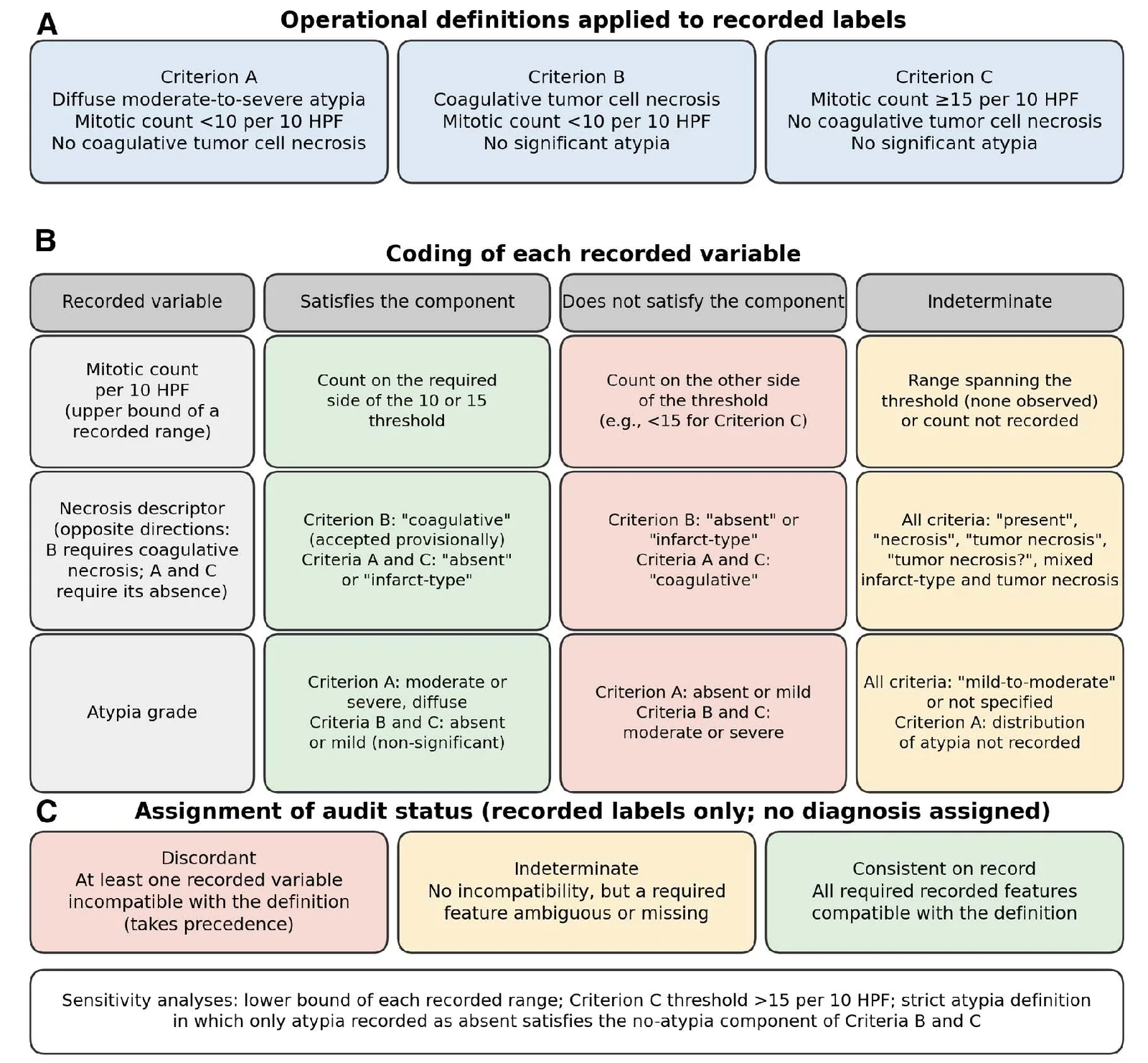
Case identification and outcome of the record-based diagnostic consistency audit. Twenty patients received an institutional diagnosis of STUMP, epithelioid STUMP, or STUMP/LMS interface between January 2017 and March 2026, and all had been managed preoperatively as presumed leiomyoma. The 18 labels recorded as Criterion A, B, or C were compared with the operational definitions stated in Section 2.3 and classified as discordant, indeterminate, or consistent on record. The epithelioid case (Case 16) and the STUMP/LMS interface case (Case 17) were described separately. Percentages refer to the 18 audited labels, and confidence intervals are exact (Clopper–Pearson). Audit statuses describe recorded labels and do not constitute diagnoses. CI = confidence interval, HPF = high-power field, LMS = leiomyosarcoma, STUMP = smooth muscle tumor of uncertain malignant potential.

### 3.2. Recorded pathological categories and mitotic counts

The recorded institutional categories were Criterion B in 9 cases (45.0%), Criterion C in 7 (35.0%), Criterion A in 2 (10.0%), epithelioid STUMP in 1 (5.0%), and STUMP/LMS interface in 1 (5.0%) (Table 2). A mitotic count was available in 19 cases and was missing only in the epithelioid case. When the upper bound of each range was used, the median count was 5 per 10 HPF (range, 1–40); 9 cases had fewer than 5 mitoses per 10 HPF, seven had 5 to 9, one had 10 to 14, and 2 had 15 or more, namely Case 7 (15 per 10 HPF) and Case 17 (30–40 per 10 HPF). Five counts were recorded as ranges: 4 to 5 in Case 3, 30 to 40 in Case 17, 3 to 5 in Case 18, 6 to 7 in Case 19, and 2 to 3 in Case 22. None of these ranges crossed the threshold of 10 or 15 mitoses per 10 HPF. When the lower bound of each range was used instead, the median fell to 3 per 10 HPF (range, 1–30), and Cases 3 and 18 moved from the stratum of 5 to 9 mitoses into the stratum below 5, which then contained 11 cases, leaving five cases in the stratum of 5 to 9.

**Table 2:** Recorded pathological categories, mitotic counts, and summary of the record-based diagnostic consistency audit (N = 20; audited A–C labels, n = 18).

Variable	Value or audit finding
Recorded institutional category	Criterion B, 9; Criterion C, 7; Criterion A, 2; epithelioid STUMP, 1; STUMP/LMS interface, 1
Mitotic count availability	Available in 19 of 20 cases; missing in the epithelioid case
Mitotic count per 10 HPF, upper bounds of ranges	Median 5 (range, 1–40); <5, 9 cases; 5–9, 7; 10–14, 1; ≥15, 2; missing, 1
Counts recorded as ranges	Case 3 (4–5), Case 17 (30–40), Case 18 (3–5), Case 19 (6–7), and Case 22 (2–3); none crossed the threshold of 10 or 15
Lower-bound sensitivity analysis	Median 3 (range, 1–30); <5, 11 cases; 5–9, 5; 10–14, 1; ≥15, 2; no audit status changed
Recorded Criterion C labels	Cases 3, 5, 8, 10, 19, 24, and 26; all had mitotic counts < 15 per 10 HPF (7/7; 95% CI, 59.0–100.0%)
Discordant A–C labels	10/18 (55.6%; 95% CI, 30.8–78.5%): Cases 1, 3, 5, 7, 8, 10, 13, 19, 24, and 26
Indeterminate A–C labels	6/18 (33.3%; 95% CI, 13.3–59.0%): Cases 2, 11, 18, 22, 23, and 25
A–C labels consistent on record	2/18 (11.1%; 95% CI, 1.4–34.7%): Cases 20 and 27
Outside the A–C audit	Case 16 (epithelioid morphology) and Case 17 (STUMP/LMS interface)
Stricter Criterion C threshold (>15 instead of ≥ 15 per 10 HPF)	No audit status changed
Strict atypia definition (only absent atypia accepted for Criteria B and C)	Discordant 14/18 (77.8%; 95% CI, 52.4–93.6%); indeterminate 2/18 (Cases 2 and 23); consistent on record 2/18; Cases 11, 18, 22, and 25 changed from indeterminate to discordant
Slide-level review indicated	All 16 discordant or indeterminate A–C labels and Case 16 (incomplete morphological documentation); Case 17 requires separate diagnostic reconciliation

Audit statuses refer to the operational definitions stated in Section 2.3, not to definitive diagnoses. Necrosis recorded as coagulative was accepted provisionally; nonspecific, uncertain, or mixed necrosis terms were coded as indeterminate and were not assumed to represent coagulative tumor cell necrosis. CIs are exact (Clopper–Pearson).

CI = confidence interval, HPF = high-power field, LMS = leiomyosarcoma, STUMP = smooth muscle tumor of uncertain malignant potential.

### 3.3. Record-based diagnostic consistency audit

All 7 labels recorded as Criterion C (Cases 3, 5, 8, 10, 19, 24, and 26) were accompanied by mitotic counts below 15 per 10 HPF (7/7; 95% CI, 59.0–100.0%), so that none was supported by the defining mitotic feature of the category. Both labels recorded as Criterion A were also discordant: Case 7 had 15 mitoses per 10 HPF with mild-to-moderate atypia, and Case 13 had 12 mitoses per 10 HPF, necrosis recorded as present, and absent atypia. Among the 9 Criterion B labels, Case 1 was discordant because moderate atypia had been recorded; Cases 2, 18, 23, and 25 were indeterminate because necrosis was described in nonspecific, mixed, or uncertain terms, with atypia additionally unspecified in Case 23; Cases 11 and 22 were indeterminate because atypia was graded as mild-to-moderate; and only Cases 20 and 27 were consistent with Criterion B on the recorded variables. Case 16, recorded as epithelioid STUMP without a mitotic count, and Case 17, recorded as STUMP/LMS interface, lay outside the A–C denominator. Overall, 10 of the 18 audited labels were discordant (55.6%; 95% CI, 30.8–78.5%), six were indeterminate (33.3%; 95% CI, 13.3–59.0%), and 2 were consistent on record (11.1%; 95% CI, 1.4–34.7%). These statuses were unchanged when the lower bounds of recorded ranges were used and when the Criterion C threshold was raised to more than 15 mitoses per 10 HPF. The classification of mild and mild-to-moderate atypia had a larger effect: under the strict atypia definition, 14 of the 18 labels were discordant (77.8%; 95% CI, 52.4–93.6%), 2 remained indeterminate (Cases 2 and 23), and the same 2 labels remained consistent on record, because Cases 11, 18, 22, and 25 changed from indeterminate to discordant. The 7 Criterion C discordances were unaffected by every sensitivity analysis, no case received a new pathological diagnosis, and the case-level results are presented in Table 3 and summarized in Figure 2.

### 3.4. Immunohistochemical findings

Immunohistochemical reporting was incomplete and heterogeneous. Quantitative or semi-quantitative Ki-67 results were available in 4 cases, with indices of 1% in Case 10, 10% in Case 16, <5% in Case 22, and 60% in Case 17. Staining for p16 was recorded as positive in Case 17 and as negative in Case 22. In 4 cases, p53 was described in the historical records as wild-type or negative, and these descriptors were retained without independent reassignment of the staining pattern. Desmin and smooth muscle actin were positive wherever they were reported, and no loss of fumarate hydratase was documented in the interpretable records. Because testing was selective and outcome events were rare or uncertain, no association between any biomarker and outcome was estimated.

### 3.5. Follow-up and oncological outcomes

A numerical follow-up duration was recorded for 17 patients (Table 4), among whom the mean follow-up was 21.3 ± 20.2 months and the median 12 months (range, 2–62). Recurrence or progression status was known for 17 patients and unknown for 3 (Cases 19, 22, and 24). Sixteen patients had no recorded recurrence or progression during a median follow-up of 11 months (range, 2–62). Case 17, whose index specimen showed 30 to 40 mitoses per 10 HPF, p16 positivity, and a Ki-67 index of 60%, underwent hysterectomy with bilateral salpingo-oophorectomy and pelvic and para-aortic lymph node dissection 2 months after the index myomectomy and was referred to medical oncology with a diagnosis of LMS. This event was recorded separately as a STUMP/LMS interface event and not as a recurrence following an independently verified STUMP diagnosis. No pregnancy after uterus-sparing index surgery was documented, and the single myomectomy performed at cesarean delivery (Case 11), which represents tumor removal at the time of birth, was not counted as a posttreatment pregnancy.

**Table 4:** Follow-up and oncological outcomes (N = 20).

Outcome variable	Value
Numerical follow-up duration available	17/20 patients
Follow-up among patients with a numerical duration, months	Mean 21.3 ± 20.2; median 12; range 2–62
Recurrence or progression status known	17/20 patients
Recurrence or progression status unknown	3/20 patients: Cases 19, 22, and 24
No recorded recurrence or progression	16 patients; median follow-up 11 months (range, 2–62); descriptive observation, not an estimate of recurrence risk
STUMP/LMS interface event	Case 17: 30–40 mitoses per 10 HPF, p16 positive, Ki-67 60%; managed as LMS 2 months after the index myomectomy; not counted as a confirmed STUMP recurrence
Pregnancy after uterus-sparing index surgery	None documented; Case 11 underwent myomectomy at cesarean delivery and was not counted as a posttreatment pregnancy

Values are mean ± SD, median, and range where indicated. A numerical follow-up interval and a known oncological status were recorded as separate variables, and the availability of one was not taken to imply the other; unknown outcomes were not counted as recurrence-free.

HPF = high-power field, LMS = leiomyosarcoma, SD = standard deviation, STUMP = smooth muscle tumor of uncertain malignant potential.

## 4. Discussion

### 4.1. Principal findings

In this institutional cohort, STUMP was not recognized before surgery, and every patient was operated on for a presumed leiomyoma. This finding describes the diagnostic pathway of the cohort; it does not show that STUMP is clinically indistinguishable from leiomyoma, because the study contains no leiomyoma comparator and cannot estimate the sensitivity or specificity of any clinical or imaging feature. The observation is consistent with earlier STUMP series in which the diagnosis was likewise established postoperatively,^[10,12,16,24,25]^ and with a report in which intraoperative frozen section agreed with the final diagnosis in only 3 of 14 patients.^[12]^ Imaging studies comparing uterine sarcoma with leiomyoma provide useful context,^[26]^ but they address the distinction between sarcoma and leiomyoma rather than the recognition of STUMP.

The principal finding of the study concerns the relationship between recorded labels and the variables recorded to support them. None of the 7 Criterion C labels was accompanied by the mitotic activity that defines the category, and across all 18 audited labels, 10 were discordant and six were indeterminate under the stated definitions. Taken together, these 16 labels identify records that require clarification; they do not represent 16 misdiagnoses. Features assessed on the original slides may have been omitted, abbreviated, or transcribed inconsistently when the reports were written or when the data were abstracted, and a record-based audit cannot determine which of these explanations applies in a given case. What the audit does establish is that, on the available documentation, the category distribution of this cohort cannot be accepted at face value. The point is not merely semantic, because the interpretation of subtype distribution and recurrence, the counseling of patients who wish to preserve fertility, and any comparison with external series all rest on the correct assignment of the underlying criteria.

### 4.2. Implications for pathological classification and reporting

The A to C categories used here are operational definitions for a record audit. They are neither an exhaustive taxonomy of STUMP nor a substitute for contemporary morphological assessment, and their value lies in making the comparison explicit and reproducible, which in turn prevents the unwarranted inference that a label incompatible with this scheme must be diagnostically wrong. The sensitivity analyses clarify which conclusions depend on the operational choices. The Criterion C result is robust, since it persisted with a stricter mitotic threshold, was unaffected by the recorded ranges, none of which crossed a threshold, and did not depend on how mild or mild-to-moderate atypia was classified. The overall proportion of discordant labels, by contrast, depended materially on the treatment of mild and mild-to-moderate atypia and rose from 10 to 14 of 18 when only absent atypia was accepted as nonsignificant, while the 2 labels consistent on record were unchanged. That proportion should therefore be read as reflecting a definitional choice and not as a misdiagnosis rate.

Necrosis terminology was the most frequent source of uncertainty. A report that states only “tumor necrosis” or “necrosis present” does not establish coagulative tumor cell necrosis, and mixed infarct-type and tumor necrosis cannot be resolved without examining the slides; every such description was therefore coded as indeterminate, and no case with ambiguous necrosis was assigned to a necrosis-driven category. The descriptor “coagulative” was itself accepted only provisionally, so that even the 2 labels consistent on record remain dependent on the accuracy and completeness of the source documentation. A series in which slides were reviewed has distinguished coagulative tumor cell necrosis explicitly from hyaline and uncertain-type necrosis,^[12]^ and the same typing, together with the grade and distribution of atypia and the field area used for mitotic counting, would have rendered most of the indeterminate statuses in this cohort unnecessary.

Slide-level gynecological pathology review should accordingly address all 16 discordant or indeterminate A to C labels and the incompletely documented epithelioid case, and the questions to be answered differ from case to case. In Cases 7 and 13, mitotic activity needs to be assessed together with the extent and grade of atypia and the nature of any necrosis; in Cases 24 and 26, the distribution of moderate-to-severe atypia requires clarification; and the scant defining features recorded for Case 10 need to be reconciled with its historical label. These are questions for expert review, not proposed alternative diagnoses. Case 17 warrants separate reconciliation, because its high mitotic count and Ki-67 index were followed by management as LMS within 2 months of the index surgery. The experience of other centers indicates that expert review can change a meaningful proportion of diagnoses,^[10–12]^ and until such review has been completed, the cohort should not be regarded as a pathologically homogeneous STUMP series or pooled with reviewed series in analyses of subtype distribution or outcome.

### 4.3. Surgical and fertility-preserving management

Nine patients underwent uterus-sparing procedures in a cohort managed preoperatively for presumed leiomyoma. This proportion describes surgical practice before the postoperative diagnosis was known; it is not a comparison of strategies selected for confirmed STUMP, and the short and incomplete follow-up, the unknown outcomes in 3 patients, the heterogeneity of the pathological categories, and the absence of any documented posttreatment pregnancy preclude inference about oncological safety or reproductive benefit. Dedicated fertility-sparing cohorts have reported reproductive and oncological outcomes after conservative management in selected patients,^[16–18]^ a pooled analysis of 9 series comprising 327 patients found no difference in overall recurrence between myomectomy and hysterectomy, although recurrences as STUMP were more frequent after myomectomy,^[27]^ and the largest multicenter comparison to date, of 401 women, reported recurrence in 12.5% over a median follow-up of 51 months.^[28]^ In that cohort, recurrence-free survival was shorter after fertility-sparing surgery when benign recurrences were counted (hazard ratio, 2.39; 95% CI, 1.36–4.19). The difference was no longer significant once benign recurrences were excluded (hazard ratio, 1.74; 95% CI, 0.90–3.34). Overall survival was similar, and conservative management was regarded as reasonable only with counseling about recurrence risk, diagnostic uncertainty, and the need for long-term surveillance.^[28]^ None of this external evidence remedies the limitations of the present series. Two individual observations require equally careful interpretation. The myomectomy performed during cesarean delivery represents removal of a tumor at the time of birth, not conception after STUMP treatment, and is reported separately for that reason. Case 25, the only nulliparous patient, underwent myomectomy and had no recorded recurrence at 48 months; this is a single observation of recurrence-free follow-up and does not constitute a fertility outcome attributable to treatment.

Morcellation status was not documented consistently, so its relationship with outcome could not be examined. Current U.S. Food and Drug Administration recommendations restrict laparoscopic power morcellation to appropriately selected patients and to procedures performed with a tissue containment system,^[19]^ outcomes after morcellation of STUMP have been described in case series,^[20]^ a multicenter cohort of 87 patients found that tumor fragmentation or morcellation was among the features associated with a higher risk of recurrence,^[29]^ and current European guidelines address the management of uterine sarcomas more broadly.^[21]^ These recommendations concern clinical practice in general. Morcellation was not an intervention evaluated in this cohort, and the present data neither support nor contradict them. Future series would be more informative if the method of tissue extraction were recorded prospectively for every case.

### 4.4. Immunohistochemistry and the interface case

Case 17 combined a high recorded mitotic count, a Ki-67 index of 60%, and p16 positivity, and was subsequently managed as LMS. This combination warrants pathological reconciliation, but a single case cannot establish the prognostic performance of any marker, and the published evidence should be represented precisely. In a meta-analysis, altered P53 and P16 expression was significantly associated with disease-free survival in STUMP, whereas Ki-67 was not, and neither marker was considered suitable as a stand-alone prognostic test^[14]^; a separate pooled analysis did not show that atypical mitotic figures increased the risk of recurrence.^[15]^ Individual retrospective series and a multicenter cohort proposing additional adverse parameters, among them tumor fragmentation, epithelioid features, a high mitotic count, a Ki-67 index above 20%, low progesterone receptor expression, and diffuse p16 expression,^[13,29]^ should be read against this pooled evidence. The historical records impose a further constraint. A result documented only as P53-negative cannot be equated retrospectively with a verified wild-type pattern, because the distribution and intensity of staining and the behavior of internal controls were not systematically available for independent interpretation, and complete absence of staining may itself represent an aberrant pattern. Likewise, a record of p16 positivity does not establish diffuse, block-type staining. The immunohistochemical data of this cohort, limited in extent and heterogeneous in method, therefore support description only.

### 4.5. Strengths and limitations

The study provides a transparent, case-level examination of the relationship between historical diagnoses and the variables recorded to support them. Its principal strengths are the retention of every original label alongside a separately reported audit status, the explicit statement of operational definitions and decision rules that allows the audit to be reproduced from Table 3, the use of sensitivity analyses to show which conclusions depend on definitional choices, and the separation of unknown outcomes from recurrence-free follow-up. Because the approach requires only variables that pathology reports routinely contain, it can also be applied in other institutions before archival STUMP cases are pooled.

Several limitations constrain interpretation. The cohort is small, retrospective, and drawn from a single center, and it includes no leiomyoma comparator. Case ascertainment depended on institutional diagnostic coding, so tumors recorded under other diagnoses would not have been identified. Pathology variables were not reexamined on slides; under-documentation may have inflated the apparent frequency of discordance or indeterminacy, whereas erroneous recorded values may have remained undetected, and the direction of the resulting bias therefore cannot be specified for individual cases. The audit is post hoc, and its decision rules were formalized after the case-level variables had been examined. The field area for mitotic counting was not documented, which prevented standardization of the counts, and the distribution of atypia was incompletely recorded. The operational A–C scheme is not exhaustive, and its overall discordance proportion varied with the definition of significant atypia, so it cannot be interpreted as a misdiagnosis rate. Immunohistochemical testing and the documentation of morcellation were heterogeneous. Follow-up was short or incomplete, and missing outcome data may conceal subsequent events. Recurrence may occur years after hysterectomy,^[7]^ and in 1 reviewed series, it occurred 52 months after diagnosis.^[11]^ A multicenter cohort reported a mean interval to recurrence of 79 months,^[29]^ and recent series have described recurrences at a median of 36 months after surgery^[30]^ and at 3, 7, and 13 years,^[31]^ far beyond the median follow-up of this cohort; the absence of recorded recurrence therefore establishes neither a low risk of recurrence nor long-term safety. Finally, the findings characterize the records of 1 institution and should not be generalized as population estimates.

## 5. Conclusions

In this cohort, STUMP was not recognized before surgery for presumed leiomyoma. The record-based audit found no mitotic-count support for any of the 7 Criterion C labels, and under the stated operational definitions, 10 of the 18 A to C labels were discordant and six were indeterminate, the number of discordant labels rising to 14 when only absent atypia was accepted as nonsignificant. These findings identify documentation-level discordance and a need for slide-level gynecological pathology review, not proven diagnostic error, and the affected cases should remain provisional until that review has been completed. Future reports would be more informative if they distinguished historical diagnoses from audit findings, specified the type of necrosis and the grade and distribution of atypia, documented the field area used for mitotic counting, and separated known from unknown outcomes. The present data do not support estimates of recurrence risk, comparative treatment effectiveness, reproductive benefit, or biomarker-based prognosis.

## Author contributions

**Conceptualization:** Firat Ekmez.

**Data curation:** Firat Ekmez.

**Formal analysis:** Firat Ekmez.

**Investigation:** Firat Ekmez.

**Methodology:** Numan Cim.

**Supervision:** Numan Cim.

**Validation:** Numan Cim.

**Writing – original draft:** Firat Ekmez.

**Writing – review & editing:** Numan Cim.
